# Correlation analysis of eye and lip canting correction after bimaxillary orthognathic surgery and orthodontic treatment

**DOI:** 10.1186/s40902-025-00493-6

**Published:** 2025-11-14

**Authors:** Haemin Kim, Dong-Woo Kim, Jaeyoung Ryu, Seunggon Jung, Hong-Ju Park, Min-Suk Kook

**Affiliations:** Chonnam National University, Gwangju, Korea, Republic of

**Keywords:** Facial asymmetry, Eye canting, Lip canting, Chin deviation, Commissure height difference, Orthognathic surgery, Orthodontic treatment

## Abstract

**Background:**

Facial asymmetry is a frequent indication for orthognathic surgery. Vertical discrepancies such as eye and lip canting strongly influence perceived facial balance. While orthognathic surgery effectively repositions skeletal structures, the extent of soft tissue canting correction remains uncertain.

**Methods:**

This retrospective study included 25 patients who underwent bimaxillary orthognathic surgery followed by orthodontic treatment between 2017 and 2023. Patients were classified into three groups: eye + lip canting (Group A, *n* = 10), Eye canting, lip canting, soft tissue chin deviation, and commissure height difference were measured using standardized frontal photographs and cephalometric radiographs at three time points: preoperative (T0), postoperative (T1), and post-debonding (T2). One-way ANOVA and Tukey’s post hoc test were used.

**Results:**

Orthognathic surgery significantly improved both eye and lip canting (*P* < 0.05). Lip canting showed greater responsiveness than eye canting. Soft tissue chin deviation and commissure height difference also demonstrated significant postoperative improvements that were maintained through the debonding stage. Minimal or no further changes occurred during orthodontic treatment. Group A demonstrated the greatest overall improvement.

**Conclusion:**

Bimaxillary orthognathic surgery effectively improves soft tissue canting, particularly of the in the lower face. Most correction occurs during the surgical phase, while orthodontics contributes minimally.

## Background

Facial symmetry is a fundamental determinant of aesthetic and functional harmony in patients undergoing orthognathic and orthodontic treatment [1]. Among elements contributing to facial integrate, vertical asymmetries such as eye and lip canting are particularly noticeable and strongly influence perceived facial attractiveness [2, 3].

Bimaxillary orthognathic surgical procedure is the primary approach for correcting skeletal discrepancies that facial asymmetry. reliably repositions hard tissues, the extent to which soft tissue canting—particularly around the lips and eyes—responds to surgical correction remains controversial [4, 5]. Postoperative orthodontic treatment refining occlusion soft tissue canting is not well [6, 7].

Lip canting is recognized as a reliable external marker of mandibular asymmetry [8, 9] and several studies have demonstrated its high responsiveness to skeletal repositioning [10, 11]. Conversely, eye canting is influenced by more complex factors, including orbital anatomy, periorbital muscle tone, and head posture [12, 13], and may show minimal improvement even after skeletal correction14.

Despite three-dimensional imaging and simulation-based planning, few studies have quantitatively the effects of orthognathic surgical procedure and orthodontic treatment on soft tissue canting [14, 15]. Furthermore, the sequence and timing of these changes—from preoperative to postoperative and post-debonding phases—remain underexplored.

This study aim to (1) evaluate changes in eye and lip canting across three treatment stages (preoperative, postoperative, and post-debonding); (2) compare the magnitude of improvement between surgical and orthodontic phases; and (3) identify different responsiveness between eye and lip canting. the relative contributions of surgical procedure and orthodontic in corrective facial asymmetry and Clinical decision-making.

## Methods

This retrospective study included 25 patients who underwent bimaxillary orthognathic surgical procedure postoperative orthodontic at Chonnam National University Hospital between 2017 and 2023. All patients exhibited clinically evident eye lip canting at initial diagnosis. This study was approved by the institutional review board of Chonnam National University Dental Hospital (CNUDH-2025-021).

Inclusion and exclusion criteria.

Inclusion criteria were:

1. clinically diagnosable eye, lip canting, or both;
2. no history of ophthalmologic disease or trauma affecting ocular inclination;
3. absence of systemic diseases influencing soft tissue symmetry.

Exclusion criteria were:

Craniofacial syndromes, incomplete medical records, or prior facial surgical procedure unrelated to orthognathic correction.

Group classification.

Patients were classified into three groups based on the type of canting:

- Group A: Eye and lip canting (*n* = 10).
- Group B: Eye canting only (n = 9).
- Group C: Lip canting only (n = 6).

Clinical assessment and measurement.

Standardized frontal photographs in natural head position (NHP) were used for canting measurement.

**Fig. 1:**
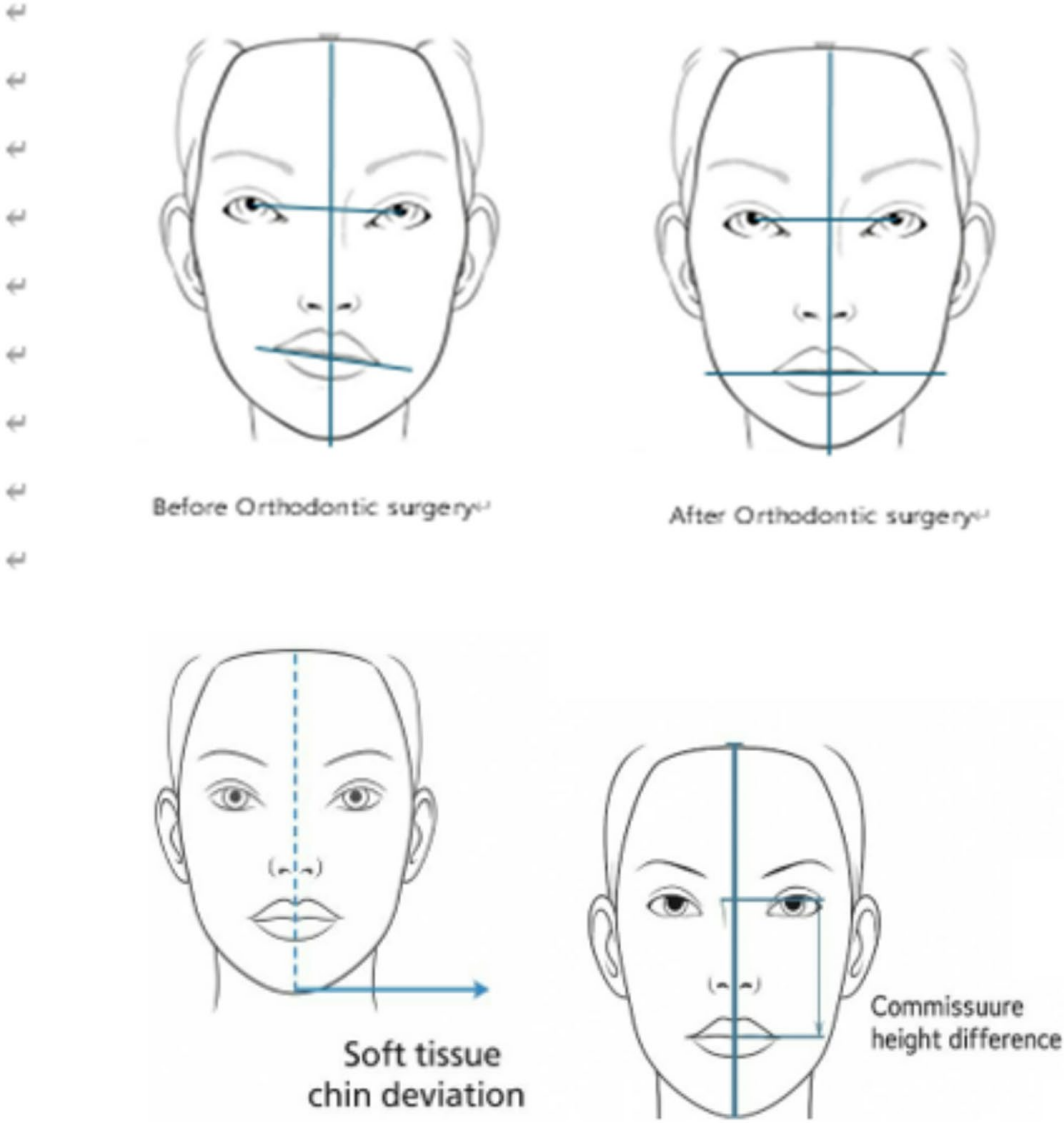
Clinical measurement methods for eye canting, lip canting, soft tissue chin deviation, and commissure height difference. Standardized frontal photographs in natural head position used to assess canting. (**A**) Eye canting: the angle between the interpupillary line and true horizontal line. (**B**) Lip canting: the angle between the interpupillary line and the line connecting both oral commissures (**C**) Soft tissue chin deviation: the horizontal distance from the soft tissue pogonion to the facial midline. (**D**) Commissure height difference: the vertical difference between the right and left oral commissures

Eye canting was defined as the angle between the interpupillary line and the true horizontal reference line. (Fig. 1)

Lip canting was measured as the angle between the interpupillary line and the line connecting both oral commissures.

Additionally, soft tissue chin deviation was measured as the horizontal distance of the chin point from the facial midline, and commissure height difference was defined as the vertical difference between the right and left oral commissures. (Fig. 1)

Measurements were performed using digital protractors in image analysis software.

Radiographic evaluation.

Skeletal movement and treatment progress were assessed lateral cephalometric radiographs at three: preoperative, postoperative (within 1 month of), and post-debonding. analysis with V-Ceph software (version 7.0, Osstem Implant Co., Ltd., Seoul, Korea).

### Statistical analysis

Data were analyzed using Statistical Package for the Social Sciences (SPSS) software (version 23.0, IBM Corp., Armonk, NY, USA). One-way analysis of variance (ANOVA) compared canting measurements across the three stages, and Tukey’s Honestly Significant Difference (HSD) test was applied for post-hoc analysis. Statistical significance was set at *P* < 0.05.

## Results

A total of 25 patients were included. No significant differences were found among Groups A, B, and C in age, sex distribution, or total treatment duration (Table 1). Baseline characteristics were deemed comparable, supporting valid intergroup comparisons

**Table 1:** Demographic characteristics of the study population

Variable	Group A (*n* = 10)	Group B (*n* = 9)	Group C (*n* = 6)	*P*-value
Age (years)	24.6 ± 3.1	25.1 ± 2.8	24.9 ± 3.5	0.87
Sex (male: female)	4:6	5:4	3:3	0.91
Treatment duration (months)	18.2 ± 2.3	17.9 ± 2.0	18.5 ± 1.8	0.78

Group A: Eye + Lip Canting; Group B: Eye Canting Only; Group C: Lip Canting Only

At baseline, no significant differences are observed among the three groups in age, sex distribution, or treatment duration

**Fig. 2:**
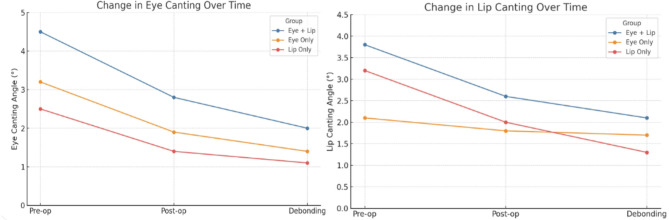
Canting angle changes across treatment stages. Comparison of eye and lip canting angles at three time points: preoperative, postoperative, and post-debonding. Lip canting shows the most substantial postoperative improvement, whereas orthodontic treatment demonstrates minimal effect

**Fig. 3:**
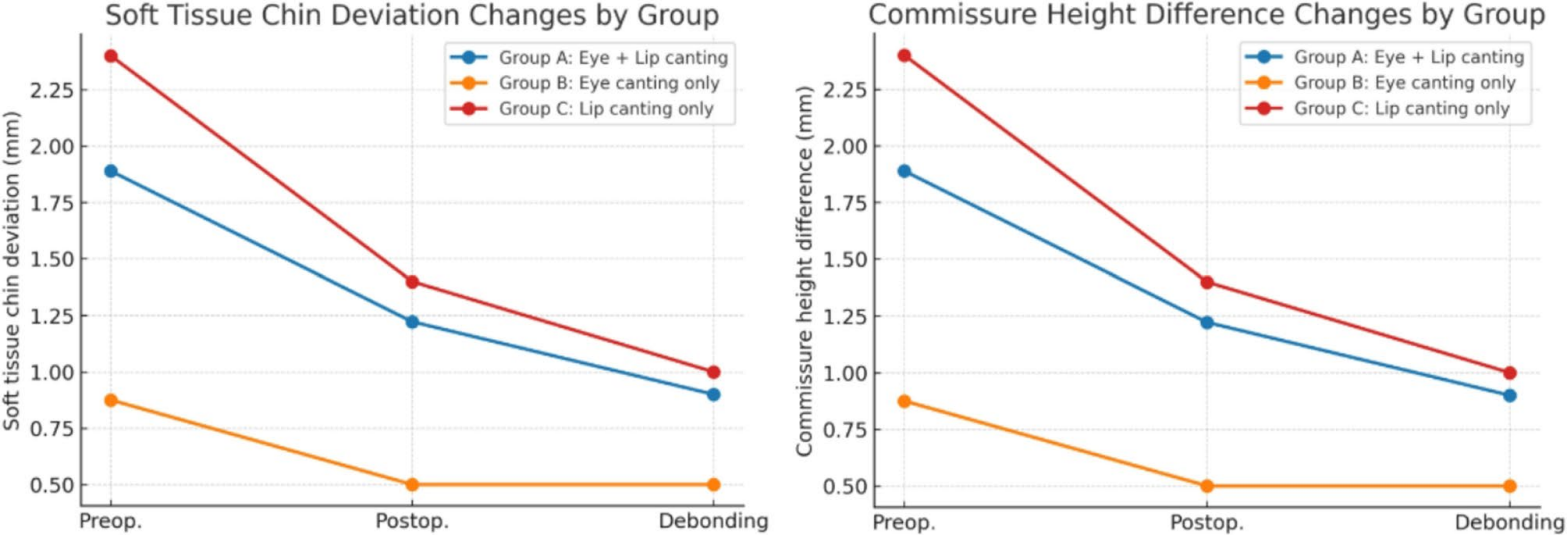
Soft tissue chin deviation & Commissure height difference changes across treatment stages. Comparison of soft tissue chin deviation and commissure height difference at three time points: preoperative, postoperative, and post-debonding. Both parameters showed marked improvement after orthognathic surgery, with stability maintained during orthodontic treatment. Group A exhibited the greatest overall reduction, whereas Groups B and C showed more limited but consistent improvements

Orthognathic surgery produced significant improvements in both eye and lip canting. (Fig. 2) In addition to eye and lip canting, orthognathic surgery also significantly reduced soft tissue chin deviation and commissure height difference, with improvements maintained throughout postoperative and debonding phases. (Fig. 3)

Group A (eye + lip canting): both angles significantly decreased postoperatively (*P* < 0.05), with lip canting showing a slightly greater reduction.

Group B (eye canting only): eye canting improved substantially postoperatively (*P* < 0.05), with no further change after debonding.

Group C (lip canting only): lip canting demonstrated a considerable decrease postoperatively and remained stable following orthodontics.

Across all groups, minimal or non-significant changes occurred during orthodontics, indicating that most soft tissue canting correction followed skeletal repositioning.

**Fig. 4:**
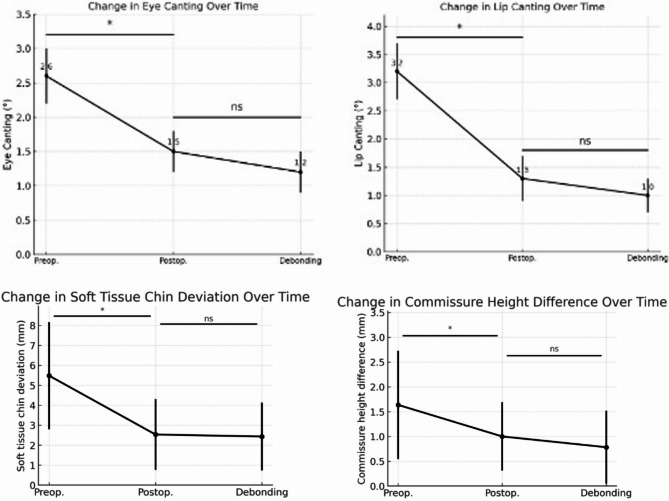
Group-wise correction trends for eye canting, lip canting, soft tissue chin deviation, and commissure height difference. Differences in correction patterns were observed among Group A (eye + lip canting), Group B (eye canting only), and Group C (lip canting only). Eye and lip canting showed the greatest postoperative improvement, particularly lip canting in Group A. Soft tissue chin deviation and commissure height difference also decreased significantly after surgery, with stability maintained during orthodontic treatment. Overall, Group A exhibited the most substantial improvements across all parameters, whereas Groups B and C demonstrated more limited but consistent changes

Group A showed the most significant overall reduction in eye and lip canting. (Fig. 4)

Group B improved only in eye canting, with orthodontics showing no additional effect.

Group C confirmed that lip canting responds more favorably to mandibular repositioning, with improvements persisting until the final follow-up.

These findings collectively indicate that orthognathic surgical procedure is the primary canting correction, particularly lip canting, orthodontic soft tissue symmetry.

## Discussion

**Fig. 5:**
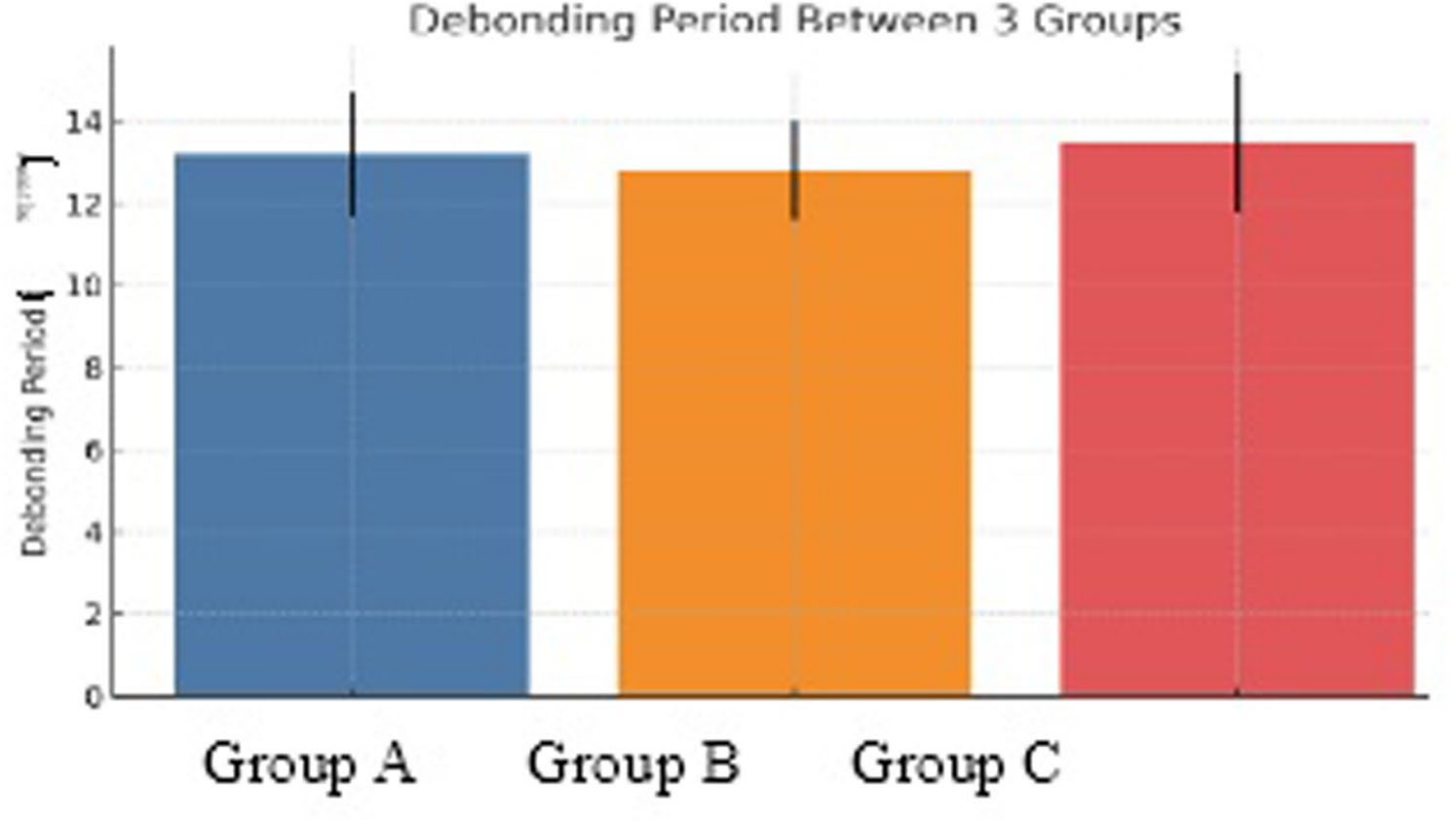
Summary of surgical and orthodontic contribution. llustrative summary that most soft tissue canting correction occurs immediately following orthognathic surgical procedure. Orthodontic treatment contributes marginally to further improvement, especially lip canting

This study investigated the impact of skeletal realignment on soft tissue canting, particularly in the perioral region [3]. The between mandibular asymmetry and lip canting has been demonstrated [13] surgical correction of lip canting generally more predictable than of eye canting [1]. This the anatomical and muscular attachments of the lips to the mandible [16]. Eye canting is influenced by orbital symmetry and neuromuscular control, making it less responsive to orthognathic surgical procedure alone [14]. Our findings that soft tissue correction occurs during the surgical phase [17] orthodontic primarily dental alignment minimal on vertical facial asymmetry [18]. (Fig. 5) Furthermore, reductions in soft tissue chin deviation and commissure height difference were observed, reinforcing the notion that orthognathic surgery exerts a significant influence on lower facial soft tissue balance. These improvements highlight that not only angular canting but also midline and vertical soft tissue parameters should be considered when evaluating treatment outcomes.

Preoperative planning and simulation enhance the surgical predictability of soft tissue changes [8]. Photographic assessment in the natural head positions essential for reliable canting measurement [19]. Persistent eye canting may require secondary interventions fat grafting or botulinum toxin injections [5]. Patients with severe lip canting showed greater postoperative improvement, suggesting a proportional asymmetry [11]. Despite technological advances, predict eye canting changes remains limited [20]. Perception of facial symmetry post may psychological adaptation rather than anatomical correction alone [21]. Some evidence that facial integrate perception is more influenced by the mid- and lower-third than by the orbital region [22].

Surgical repositioning of the maxillomandibular complex indirectly affects soft tissues of the lower face [2]. Multiple imaging modalities, including cone-beam computed tomography (CBCT) and stereophotogrammetry, facilitate detailed soft tissue analysis [23]. Our findings are consistent with prior research indicating greater predictability of soft tissue changes in the lower face compared with the upper face [15]. The limited improvement in eye canting underscores the importance of setting realistic treatment expectations [24]. Stability of lip canting correction is generally high when precise surgical techniques are applied [4]. However, residual asymmetry may still influence postoperative satisfaction despite objective improvements in facial balance [7]. Multidisciplinary collaboration remains essential for optimal management of facial asymmetry [25].

Further research should investigate long-term outcomes using objective aesthetic indices and patient-reported outcome measures (PROMs) [26]. Differences in aging patterns between the upper and lower face may influence long-term canting perception [27]. Advanced planning tools enable surgeons to visualize skeletal and soft tissue movements preoperatively [28]. Analysis of our expanded cohort also revealed variability in soft tissue response depending on skeletal movement vectors [10]. Quantitative evaluation using cephalometric and photographic data provides a robust foundation for future comparative studies [6]. Lip canting correction enhances smile esthetics and is associated with higher patient satisfaction scores [12]. However, eye asymmetry has a more profound effect on gaze perception than overall facial symmetry [29].

Some studies emphasize that chin and midline deviations correlate more strongly with perceived facial asymmetry than eye canting [30]. Limitations of our study include sample size and reliance on 2D analysis, which should be addressed in future research [9]. Our findings reinforce the hypothesis that lip canting, due to its direct association with mandibular repositioning, is more predictable than eye canting [4]. Biomechanical factors such as the orientation of masseter and mentalis musculature may amplify soft tissue correction in the lower third of the face [5]. The deeper skeletal structures of the orbital region and the periorbital muscles may not be fully impacted by maxillary repositioning alone [6]. Furthermore, the skin and subcutaneous tissues in the periorbital area are less elastic and mobile than those in the perioral region [7]. Psychosocial that patients tolerate upper facial asymmetry than discrepancies near the mouth and chin, which are more noticeable during speech and expression [8].

Patient satisfaction is more closely to midface and lower face improvements than subtle orbital changes, as supported by several facial esthetics studies [9]. Preoperative 3D virtual planning significantly improved predictability lip canting correction but eye canting [10]. Cases, upper facial asymmetry require secondary esthetic procedures such as blepharoplasty or supraorbital augmentation to achieve balance [11]. Dynamic smile analysis before and after shows that soft tissue changes are more predictable and consistent than periorbital changes [12]. Given the central role of the lips in emotional expression, their asymmetry is often more disturbing to patients than eye asymmetry [13]. Neuromuscular studies suggest that the orbicularis oris and related muscles adapt to bony realignment [17], while the levator palpebrae and frontalis muscles remodel following surgery, explaining persistent eye canting [14].

Clinicians should manage expectations by discussing these anatomical limitations during preoperative counseling [15]. Patients with severe preoperative asymmetry may gain the most esthetic benefit from surgical intervention [24], consistent with prior findings that greater skeletal movement correlates with improved lower-face soft tissue asymmetry [21]. Orthodontic alignment optimizes occlusion but has minimal impact on soft tissue symmetry once skeletal discrepancy is corrected [20]. Longitudinal studies confirm that lip canting correction remains stable for up to [5] years postoperatively when combined with rigid internal fixation [19]; however, relapse may occur in cases with persistent neuromuscular imbalance or parafunctional habits [27]. Therefore, long-term follow-up and muscle training may enhance outcome stability [22].

The 3D surface scanning combined with PROMs provides more comprehensive evaluation metrics than traditional cephalometric analysis [29]. This multimodal approach allows clinicians to integrate objective and subjective treatment outcomes [23]. Our study contributes to the growing evidence favoring maxillomandibular-based correction strategies for lower-face asymmetry [28]; it underscores the need for adjunctive esthetic procedures for patients seeking complete facial harmony, especially in the upper face [25]. Several systematic reviews align with our findings, emphasizing the varying responsiveness of facial thirds to orthognathic surgery [26]. Advanced techniques such as computer-aided surgical simulation (CASS) have improved the precision of asymmetry correction [18], although further refinement of predictive algorithms remains essential, particularly for complex craniofacial deformities [30]. Future research should investigate AI-based image analysis to enhance preoperative asymmetry quantification [18].

## Conclusion

This study demonstrated that bimaxillary orthognathic surgical procedure significantly improves soft tissue canting, particularly of the lips. Lip canting, closer to the skeletal movement zone, responded more predictably than eye canting, which is by complex orbital structures and mus. Orthodontic treatment minimal soft tissue correction. These findings highlight the precise preoperative evaluation, mandibular asymmetry and lip alignment, to facial symmetry outcomes. Patients should be persistent eye canting and the limits of surgical correction in the upper face. Clinical lower facial symmetry, greater impact on esthetics and patient satisfaction. studies incorporating patient-reported outcomes are to better the objective and subjective aspect of asymmetry correction.

## Data Availability

The datasets generated and analyzed during the current study are available from the corresponding author on reasonable request.

## Abbreviations

(NHP): Natural head position
(CBCT): Cone-beam CT
(AI): Asymmetry index
(FH) plane: Frankfort horizontal
(ANOVA): Analysis of variance
(SPSS): Statistical Package for the Social Sciences
(PROMs): Patient-reported outcome measures
